# Validation of Diet History Questionnaire in Assessing Energy and Nutrient Intakes of Iranian Population

**Published:** 2019-06

**Authors:** Fatemeh TOORANG, Bahareh SASANFAR, Soodeh RAZEGHI JAHROMI, Soraiya EBRAHIMPOUR KOUJAN, Saba NARMCHESHM, Ali RAFEI, Kazem ZENDEHDEL

**Affiliations:** 1. Cancer Research Center, Cancer Institute of Iran, Tehran University of Medical Sciences, Tehran, Iran; 2. Department of Community Nutrition, School of Nutritional Sciences and Dietetics, Tehran University of Medical Sciences, Tehran, Iran; 3. Department of Clinical Nutrition and Dietetics, School of Nutrition and Food Technology, Shahid Beheshti University of Medical Sciences, Tehran, Iran; 4. Breast Diseases Research Center, Cancer Institute of Iran, Tehran University of Medical Sciences, Tehran, Iran

**Keywords:** Diet history questionnaire, Validation, Nutrient intake, Cancer

## Abstract

**Background:**

This study described validity of Diet History Questionnaire (DHQ) for assessing energy and nutrient intake among Iranian population.

**Methods:**

A group of experienced nutritionists translated the DHQ to Farsi language and modified it based on Iranian food habits and cooking methods. We recruited 244 healthy adults with a mean age of 42.83 ± 0.75 yrs. from healthy individuals who were friends or relatives of patients in the Cancer Institute of Iran from April 2011 to February 2012. We used the DHQ to assess dietary intakes through interviews as well as at least four 24-h recalls one in every season. Reliability was estimated by comparing data of DHQ with mean intake from 24-hour recalls using deattenuated and energy-adjusted Spearman correlation coefficients. We classified dietary intakes of two methods into three groups to probe if DHQ correctly allocates subjects into different intake groups compared to reference method. The results are reported as percent of disagreement, adjacent agreement, and complete agreement.

**Results:**

Deattenuated spearman correlation ranged from 0.18 for vitamin B_12_ and fat to 0.60 for sugar. It ranged from 0.13 for pantothenic acid to 0.60 for sugar in men and from 0.07 for fat to 0.58 for sugar in females. The complete agreement of methods ranged from 51% for selenium to 36% for carotene in the entire sample, from 50% for energy to 31% for niacin in males and from 49% for sugar to 27% for vitamin B_12_ in females.

**Conclusion:**

The DHQ is a valid tool for assessing most nutrients intake among Iranian population. In addition, it is a proper instrument in categorizing individuals based on their dietary intakes.

## Introduction

Cancer is reported as a common cause of death worldwide, with its burden is increasing in the low- and middle-income countries due to longer life expectancy and cultural transition in these countries(1). Nutritional exposures are major modifiable lifestyle factors for many chronic diseases, including cancers (2, 3). Although protective effects of some nutritional components like fruits and vegetables on the occurrence and prevention of different diseases have been clearly understood, scientists are not certain about most of nutritional factors in detail. This uncertainty is partly due to lack of a precise tool for intake assessment and partly related to the large variation of nutritional status in different societies (3).

On the other hand, the relationship between nutritional factors and cancer incidence is affected by genetic and environmental factors (4), highlighting the importance of national studies on eating behaviors and nutritional intake. Therefore, nutritional risk factors for cancer should be determined in every nation with a valid instrument. Moreover, as food habits and cultures are different for every nation, dietary questionnaires should be developed especially for every food culture (5).

Assessment of dietary intake is a challenging task and needs appropriate instrument (5, 6). There are several instruments for assessing food intake. The most frequently used are food records, 24-h recalls and food frequency questionnaires. Although food records and 24-h recalls are more accurate, they are too expensive. Moreover, they assess short time intake and nutritional information for longer periods is necessary for studying the association between diet and chronic disease (6). Food frequency questionnaires (FFQ) are the most frequently used tools for assessing nutritional in-take (5, 7). Their popularity stems from their easy administration and low cost. Moreover, they assess long time intake in a single administration (6).

There are some valid Iranian FFQs, but most of them have been examined in specific regions of Iran and they are not assessed for accuracy in the entire Iranian population (8–10). Moreover, FFQs usually investigate fewer details about cooking methods (11) which play an important role in tumor development. The National cancer institute of America has recently developed an FFQ-based food questionnaire named Diet History Questionnaire (DHQ) to assess seasonal intake, nutritional supplement and cooking methods in addition to food frequencies and portions sizes (12). Translation and adaptation of DHQ and assessment of its validity have been done in different countries, including Japan and Canada (13–15). However, it has not been translated in the low and middle-income countries, including Iran.

We aimed to develop a Farsi version of DHQ and validate it for epidemiological studies in Iranian population.

## Methods

### Translating and modifying the DHQ

A group of experienced nutritionists translated the DHQ of the National Cancer Institute of America to Farsi language and modified it based on Iranian food habits and cooking methods. It was achieved by adding Iranian mixed dishes and omitting or adding some fruits, vegetables or snacks. Furthermore, some portion sizes and food intake frequencies were modified based on previous studies about food intake in Iran (8, 9). The final DHQ consists of 146 main questions related to mixed dishes and food items like bread or fruits. Like the original DHQ, each main question in our questionnaire has two parts, consisting of multiple choice questions related to frequency and portion sizes. Consumption of seasonal foods like fruits is investigated in two questions, one of which asks in season and the other in other times of consumptions. Some questions were supplemented with questions related to fat content and cooking methods. The questionnaire is available on request.

### Subjects

We recruited 244 healthy adults aged 19–60 yrs. from healthy individuals who were friends or relatives of patients in the Cancer Institute of Iran from April 2011 to February 2012. Cancer Institute of Iran is a referral cancer hospital and admits patients from most part of Iran (16). Therefore, the patients in this center could be representative of the Iranian population, which makes it a reasonable place for studying cancer in Iranian. We recruited individuals from different age groups in 5-yr strata. All the individuals were interviewed by trained interviewers. The participants were recruited after evaluation of the study criteria and signing a written informed consent. Exclusion criteria were being on a special diet and being pregnant or lactating. Moreover, based on the study protocol, we planned to exclude subjects who reported a substantial change in diet during the study.

The study was approved by Ethical Committee of Tehran University of Medical sciences (No.11360). Interviewer explained the study procedures and obtained a written informed consent from all research subjects.

### Dietary intake assessment

Dietary intakes were assessed by an interview-based DHQ and at least four 24-h recalls. We followed up the participants for a year and each participant performed at least one interview in every season and planned to perform the interviews on at least three weekdays and one weekend for each participant in order to consider more variability in the food intake. Recalls were converted to grams per day and proper codes of food composition table were allocated to each food by trained nutritionists in accordance with uniform procedures. DHQ data were converted to gr/day in separate programs developed by authors using the STATA software. Daily food intakes of both DHQ and recalls were inverted to energy and nutrient intakes using food composition table in an access program made by authors. The Iranian food composition table covers only raw foods and limited nutrients (17). Therefore, we used the McCance and Widdowson’s Food composition table (18) and supplemented it with the Iranian counterpart (19) for some special Iranian foods. We reported medians of intake estimated by DHQ, and means of 24-h recalls. Reliability was estimated by comparing data of the DHQ with mean intakes from 24-hour recalls by Spearman correlation coefficients (5). Nutrient intakes were adjusted for energy intake by residual methods. Since the reference methods like food record or 24h-recall are subject to day to day variation in dietary intake, the correlation between FFQs and recall data is usually underestimated (20). Therefore, deattenuated correlation coefficients were estimated by Rosner and Willett’ formula to modify the within-person variations in recalls (6, 21).

The ability of the questionnaire to properly classify subjects in a group of intake is the main characteristic of FFQ-based questionnaires, which represents their validity (22, 23). Therefore, we classified subjects to tree dietary intake groups based on their DHQ and 24-h recalls. We probed if the DHQ allocated the subjects in the proper intake groups assuming that 24-h recall as the reference method. We assumed complete agreement if subjects were in the same group of intake by both methods; Adjacent agreement was if they were classified almost similar. for example, they were classified in medium intake by DHQ but in the higher intake group by 24-h recalls or vice versa; we grouped them as complete disagreement if they classified in the high intake group by DHQ and low intake group by 24-h recalls or vice versa. All statistical analyses were performed using the STATA software (Special Edition, Ver. 11.2, STATA Corp, Texas, and USA).

## Results

We interviewed 244 subjects in the study. They consisted of 138 males and 106 females with a mean age of 42.83 ± 0.75 yrs. and were moderately overweighed (mean BMI was 25.29±0.8). They were mainly married and literate (Table 1). Approximately 45 min was spent to administer the DHQ. As expected, the DHQ estimated energy and nutrient intakes higher than recall and men reported higher intake of energy than women in both methods (Table 2).

**Table 1:** Characteristics of participants in validation study of Diet History Questionnaire in Iranian population

***Characteristics***		***Total (N=244)***	***Male (N=138)***	***Female (N=106)***
Marital status (%)	Single	41 (17.8)	19 (13.77)	22(21.57)
	Married	199(82.92)	119(86.23)	80(78.43)
Education level (%)	Illiterate	7(2.95)	3(2.2)	4(3.96)
	Lower than high school	82(34.6)	42(30.88)	40(39.60)
	High school	90(37.97)	51(37.5)	39(38.61)
	Academic	58(24.47)	40(29.41)	18(17.82)

**Table 2:** Median daily intakes of energy and nutrient estimated by study methods in validation study of Diet History Questionnaire in Iranian population

***Nutrient***	***Unit***	***Mean 24-hour recalls***			***DHQ***		
		***Total***	***Male***	***Female***	***Total***	***Male***	***Female***
Energy	Kcal	1949.35	2296.79	1641.78	3068.63	3503.83	2652.81
Carbohydrate	G	286.22	347.38	239.61	397.87	472.71	328.69
Protein	G	71.02	78.57	59.70	145.31	171.74	117.11
Fat	G	57.87	60.47	54.22	97.17	103.23	90.74
Fiber	G	87.87	60.47	54.22	97.17	103.23	90.74
Sugar	G	34.70	38.99	29.13	41.20	47.97	33.93
Vitamin A	RE	292.84	631.46	513.09	1021.09	1108.23	881.06
Carotene	μg	2449.16	2663.28	2164.48	4119.87	4552.73	3769.11
Niacin	Mg	16.71	19.30	13.17	23.76	31.34	18.26
Vitamin B6	Mg	1.24	1.35	1.07	0.2	0.2	0.17
Vitamin B5	Mg	4.16	4.48	3.63	1.74	2.18	1.42
Folate	μg	236.33	273.54	203.45	487.93	566.96	423.84
Vitamin B_12_	μg	1.80	2.02	1.56	2.95	3.53	2.37
Biotin	μg	25.95	29.23	22.37	66.48	77.71	60.10
Vitamin C	Mg	122.34	136.29	104.04	232.98	256.92	207.64
Sodium	Mg	1960.27	2292.11	1568.12	2948.96	3521.03	2255.82
Potassium	Mg	2696.96	3028.81	2328.39	6644.77	7472.96	5567.91
Calcium	Mg	717.28	789.26	647.15	2199.96	2431.79	1434.77
Magnesium	Mg	222.02	255.14	183.82	477.84	552.26	418.95
Iron	Mg	18.49	22.24	18.80	20.76	24.25	16.38
Zinc	Mg	7.35	8.49	6.32	17.54	20.38	12.79
Selenium	Mg	79.89	104.08	61.19	103.54	137.42	80.75

Correlation coefficients of energy and nutrient intakes between the mean 24hour-recalls and DHQ are reported in Table 3. Crude spearman correlations between DHQ and 24-h recall ranged from 0.17 for vitamin B_12_ and fat to 0.55 for carbohydrate. As expected deattenuated spearman correlation was slightly higher than the crude and ranged from 0.18 for vitamin B_12_ and fat to 0.60 for sugar. Males showed a correlation close to the entire study population. Crude spearman correlations in males ranged from 0.12 for pantothenic acid to 0.44 for energy. Like the entire study population, deattenuated spearman correlations were slightly higher than the crude and ranged from 0.13 for pantothenic acid to 0.60 for sugar in men. Crude Spearman correlations of the two methods were slightly lower in females and ranged from 0.07 for fat to 0.47 for sugar. Deattenuation mildly increased the correlation to 0.07 for fat and 0.58 for sugar. Deattenuated spearman correlations were equal to or greater than 0.5 for energy, carbohydrate, sugar and selenium. They were greater than 0.3 for protein, fiber, niacin, pyridoxine, folate, biotin, sodium, magnesium, potassium, calcium, zinc and iron. Considering the classification of individuals based on their intake, the DHQ places most subjects in the correct class. The complete agreement of methods ranged from 36% for carotene to 51% for selenium in the entire population; disagreements were really low, from 7% for carbohydrate to 19% for vitamin B_12_. As expected, the agreements were slightly lower in subgroups. The complete agreement ranged from 31% for niacin to 50% for energy in males and from 27% for vitamin B_12_ to 49% for sugar in females. Disagreement ranged from 9% for energy to 21% for niacin in males and 13% for selenium to 25% for iron in females (Table 4).

**Table 3:** Correlation coefficients of energy and nutrient intakes between the mean 24hour-recalls and DHQ in validation study of Diet History Questionnaire in Iranian population

***Nutrient***	***Total***			***Male***			***Female***		
	***Crude***	***Deattenuated***	***Energy-adjusted***	***Crude***	***Deattenuated***	***Energy-adjusted***	***Crude***	***Deattenuated***	***Energy-adjusted***
Energy	0.51	0.52	-	0.44	0.47	-	0.23	0.24	-
Carbohydrate	0.55	0.56	0.19	0.43	0.45	0.23	0.22	0.23	0.01
Protein	0.42	0.46	0.03	0.29	0.33	0.01	0.20	0.21	0.12
Fiber	0.31	0.33	0.26	0.22	0.23	0.30	0.25	0.27	0.22
Sugar	0.46	0.60	0.56	0.40	0.60	0.57	0.47	0.58	0.58
Fat	0.17	0.18	0.19	0.24	0.25	0.21	0.07	0.07	0.03
Vitamin A	0.21	0.23	0.25	0.13	0.14	0.23	0.21	0.22	0.29
Carotene	0.20	0.22	0.27	0.17	0.18	0.24	0.22	0.25	0.31
Niacin	0.44	0.45	0.10	0.34	0.36	0.15	0.17	0.18	0.13
Vitamin B6	0.30	0.31	0.31	0.38	0.39	0.36	0.20	0.21	0.22
Vitamin B5	0.27	0.28	0.14	0.12	0.13	0.03	0.25	0.27	0.24
Folate	0.30	0.31	0.14	0.20	0.21	0.02	0.25	0.26	0.36
Vitamin B_12_	0.17	0.18	0.11	0.17	0.19	0.10	0.10	0.11	0.07
Biotin	0.29	0.31	0.21	0.27	0.29	0.29	0.11	0.12	0.14
Vitamin C	0.22	0.23	0.21	0.15	0.16	0.19	0.20	0.21	0.25
Sodium	0.44	0.46	0.15	0.32	0.34	0.07	0.23	0.24	0.27
Potassium	0.34	0.35	0.24	0.34	0.36	0.21	0.14	0.14	0.31
Calcium	0.29	0.30	0.20	0.24	0.25	0.18	0.26	0.27	0.25
Magnesium	0.39	0.40	0.19	0.41	0.42	0.15	0.12	0.13	0.24
Iron	0.39	0.40	0.05	0.34	0.39	0.04	0.37	0.39	0.03
Zinc	0.32	0.36	0.07	0.27	0.31	0.07	0.11	0.12	0.08
Selenium	0.51	0.53	0.16	0.33	0.35	0.04	0.28	0.28	0.22

**Table 4:** Percentages of agreement, adjacent agreement and complete disagreement of DHQ derived intakes and the average 24h-recalls in validation study of Diet History Questionnaire in Iranian population

***Nutrient***	***Total***			***Male***			***Female***		
	***Complete agreement***	***Adjacent agreement***	***Complete disagreement***	***Complete agreement***	***Adjacent agreement***	***Complete disagreement***	***Complete agreement***	***Adjacent agreement***	***Complete disagreement***
Energy	0.50	0.40	0.09	0.50	0.41	0.09	0.36	0.43	0.19
Carbohydrate	0.48	0.44	0.07	0.44	0.44	0.11	0.38	0.42	0.19
Protein	0.43	0.45	0.11	0.41	0.42	0.16	0.36	0.49	0.14
Fiber	0.43	0.42	0.14	0.35	0.46	0.18	0.43	0.40	0.16
Sugar	0.41	0.39	0.14	0.42	0.39	0.13	0.49	0.35	0.10
Fat	0.38	0.45	0.17	0.43	0.42	0.14	0.32	0.46	0.21
Vitamin A	0.39	0.43	0.18	0.35	0.49	0.15	0.36	0.47	0.16
Carotene	0.36	0.45	0.18	0.43	0.39	0.17	0.33	0.49	0.17
Niacin	0.45	0.42	0.12	0.31	0.48	0.21	0.33	0.49	0.17
Vitamin B6	0.45	0.42	0.13	0.41	0.49	0.10	0.44	0.37	0.18
Vitamin B5	0.43	0.41	0.15	0.37	0.44	0.18	0.42	0.40	0.17
Folate	0.43	0.44	0.13	0.44	0.39	0.16	0.37	0.48	0.14
Vitamin B_12_	0.37	0.43	0.19	0.43	0.37	0.19	0.27	0.52	0.19
Biotin	0.43	0.41	0.15	0.43	0.42	0.14	0.39	0.41	0.19
Vitamin C	0.39	0.43	0.17	0.35	0.46	0.19	0.34	0.48	0.17
Sodium	0.45	0.45	0.09	0.45	0.40	0.14	0.39	0.43	0.17
Potassium	0.44	0.43	0.12	0.39	0.43	0.17	0.39	0.40	0.19
Calcium	0.46	0.39	0.14	0.39	0.43	0.17	0.43	0.50	0.15
Magnesium	0.45	0.43	0.11	0.46	0.41	0.12	0.36	0.44	0.19
Iron	0.49	0.38	0.12	0.39	0.47	0.13	0.38	0.35	0.25
Zinc	0.43	0.44	0.13	0.41	0.44	0.14	0.38	0.40	0.21
Selenium	0.51	0.41	0.08	0.44	0.43	0.12	0.37	0.48	0.13

## Discussion

In this study, we investigated the validity of DHQ for assessing energy and nutrients intakes compared to 24-h recalls among the Iranian population. Deattenuated spearman correlation between DHQ estimated intakes and recalls ranged from 0.18 for vitamin B_12_ and fat to 0.60 for sugar. The questionnaire is a valid instrument for assessing energy, carbohydrate, sugar, and selenium and has acceptable validity for investigating the intake of protein, fiber, niacin, pyridoxine, folate, biotin, sodium, magnesium, potassium, calcium, zinc and iron. In addition, the modified DHQ is a proper instrument for categorizing individuals based on their dietary intakes, which makes it a qualified tool in epidemiological studies.

To the best of our knowledge, this is the first study to validate a Farsi version of the DHQ among the Iranian population. Previous attempts examined the validity of FFQs in Iran; the most important ones were FFQ of Tehran Lipid and Glucose Study (TLGS) and FFQ of Gastro-Esophageal Malignancies in Northern Iran (GEMINI) cohort study (8, 9).

The interclass correlation coefficients between FFQ and reference method in TLGS were higher than ours. Although the number of subjects was substantially higher in our study (243 vs. 132), they collected twelve 24-h recalls which is much more frequent than what we did (9). Increasing the number of reference method has a positive effect on the correlation between the reference method and the investigated questionnaire (24). The second was an FFQ developed especially for individuals living in Golestan, a province in north of Iran. The correlation coefficients between FFQ and recalls in this study were higher than ours and ranged from 0.49 to 0.82. The number of recalls was much higher than ours, 12 recalls in a year, collected on a monthly basis (8). Moreover, the study groups of both studies were familiar with dietary questionnaires. Both of these studies assessed the validity of FFQs in a specific region, which limits the generalizability of their results to other populations. In contrast, the subjects of our study were selected from a referral hospital in capital city of Iran, representing almost the entire Iranian population. About 40% of the patients in the Cancer Institute of Iran were referred from other provinces, indicating that the patients in the cancer institute can be seen as a cross representative of all Iranian cancer patients (16). In spite of potential reservations, we believe that research subjects of this study can be considered as a representative group for Iran and DHQ can be used in most geographical regions of Iran. This assumption could be slightly biased as it will cover the people who live in a remote area and may not visit cancer institute in the capital city of Tehran because of socioeconomic reasons.

The corresponding correlations for men in our study were similar to the entire study group and were slightly higher than those of women. Several validation studies have shown lower correlations in females (8, 9). The lower validity in women may be explained by the fact that underreporting is usually more common in females (25). However, part of this difference could be due to using same portion sizes for both sexes in questionnaires, where women report their food intake by different portion sizes. Some studies have diminished this problem by using different portion sizes for each sex or open questions for portion sizes (26).

There were some limitations in our study. The most important limitation was choosing 24-h recalls as the reference method. Recalls rely on memory, which is a source of error similar to FFQ. A great part of the literature suggests food record as the reference method (6). However, food record was not a proper method in populations with low nutritional literacy, such as Iran (8, 9).

Another limitation of our study was collecting all dietary data through interview. Interviews increase the costs compared to self-administration. However, using self-administered questionnaires was not a suitable approach in the Iranian adult population (8, 9). Self-administered questionnaires are applicable only among literate and motivated individuals who are familiar with food intake assessment. Self-administration will cause selection bias in societies with low education (20, 27). We used phone-administered 24-h recalls because our participants were living in different cities and it was not feasible to conduct face-to-face interview. This approach improved our ability to select subjects living in different parts of Iran without raising costs and selection bias. The validity of phone interview is close to that of face-to-face interview (23).

The DHQ estimated dietary intakes generally higher than 24-h recalls in our study. It is a common result in validation studies (6, 8, 9), partly due to the large food list in FFQ, and could be diminished by energy adjusting. It should be reminded that FFQ is applied to rank individuals based on their intake; therefore, shifting all individuals intake to upper level, in the same manner, would not disturb their relations with diseases (6). Moreover, underreporting has been distinguished in recalls; for example, energy intake assessed by recalls is underreported compared to doubly labeled water (28).

In epidemiologic studies that attempt to clarify the role of nutritional intake in development of diseases, individuals should be ranked based on their dietary intake. Therefore, the ability of an instrument to place the individuals in their correct intake class is an essential part of its validity (6). Hence, one of the strengths of our study is assessing the agreement of DHQ with 24-h recalls to allocate individuals to intake groups. In this regard, the disagreement was less than one-fourth of the study population, indicating that the DHQ is a suitable tool for epidemiologic studies. It was less than 16% in the validation study of TLGS FFQ (9).

## Conclusion

The DHQ demonstrated a good capacity to estimate intake of energy, carbohydrate, sugar and selenium and was properly valid for assessing protein, fiber, niacin, pyridoxine, folate, biotin, sodium, magnesium, potassium, calcium, zinc and iron. It perfectly ranked individuals based on their intake in terms of energy and most nutrients –an important characteristic that makes it suitable for epidemiologic studies, especially in case/control or cohort studies. The questionnaire and the program for analyses of nutrients are available on request.

## Ethical considerations

Ethical issues (Including plagiarism, informed consent, misconduct, data fabrication and/or falsification, double publication and/or submission, redundancy, etc.) have been completely observed by the authors.

## References

[1] Mousavi SM Gouya MM Ramazani R (2008). Cancer incidence and mortality in Iran. Ann Oncol, 20:556–563. 19073863 10.1093/annonc/mdn642

[2] e Silva GA De Moura L Curado MP (2016). The fraction of cancer attributable to ways of life, infections, occupation, and environmental agents in Brazil in 2020. Plos one, 11:e0148761. 26863517 10.1371/journal.pone.0148761 PMC4749327

[3] Kushi LH Doyle C McCullough M (2012). American Cancer Society Guidelines on nutrition and physical activity for cancer prevention: reducing the risk of cancer with healthy food choices and physical activity. CA Cancer J Clin, 62:30–67. 22237782 10.3322/caac.20140

[4] Tholin S Rasmussen F Tynelius P Karlsson J (2005). Genetic and environmental influences on eating behavior: the Swedish Young Male Twins Study. Am J Clin Nutr, 81:564–569. 15755823 10.1093/ajcn/81.3.564

[5] Cade J Thompson R Burley V Warm D (2002). Development, validation and utilisation of food-frequency questionnaires–a review. Public Health Nutr, 5:567–587. 12186666 10.1079/PHN2001318

[6] Willett W (2012). Nutritional epidemiology. Oxford University Press.

[7] Thompson FE Moler JE Freedman LS (1997). Register of dietary assessment calibration-validation studies: a status report. Am J Clin Nutr, 65:1142S–1147S. 9094911 10.1093/ajcn/65.4.1142S

[8] Malekshah A Kimiagar M Saadatian-Elahi M (2006). Validity and reliability of a new food frequency questionnaire compared to 24 h recalls and biochemical measurements: pilot phase of Golestan cohort study of esophageal cancer. Eur J Clin Nutr, 60:971–7. 16465196 10.1038/sj.ejcn.1602407

[9] Mirmiran P Esfahani FH Mehrabi Y Hedayati M Azizi F (2010). Reliability and relative validity of an FFQ for nutrients in the Tehran lipid and glucose study. Public Health Nutr, 13:654–662. 19807937 10.1017/S1368980009991698

[10] Mohammadifard N Omidvar N Houshiarrad A (2011). Validity and reproducibility of a food frequency questionnaire for assessment of fruit and vegetable intake in Iranian adults. J Res Med Sci, 16:1286–97. 22973322 PMC3430018

[11] Subar AF Thompson FE Smith AF (1995). Improving food frequency questionnaires: a qualitative approach using cognitive interviewing. J Am Diet Assoc, 95:781–788. 7797809 10.1016/s0002-8223(95)00217-0

[12] Subar AF Thompson FE Kipnis V (2001). Comparative validation of the Block, Willett, and National Cancer Institute food frequency questionnaires: the Eating at America’s Table Study. Am J Epidemiol, 154:1089–1099. 11744511 10.1093/aje/154.12.1089

[13] Csizmadi I Kahle L Ullman R (2007). Adaptation and evaluation of the National Cancer Institute’s Diet History Questionnaire and nutrient database for Canadian populations. Public Health Nutr, 10:88–96. 17212847 10.1017/S1368980007184287

[14] Flood A Subar AF Hull SG (2006). Methodology for adding glycemic load values to the National Cancer Institute Diet History Questionnaire database. J Am Diet Assoc, 106:393–402. 16503230 10.1016/j.jada.2005.12.008

[15] Millen AE Midthune D Thompson FE Kipnis V Subar AF (2006). The National Cancer Institute diet history questionnaire: validation of pyramid food servings. Am J Epidemiol, 163:279–288. 16339051 10.1093/aje/kwj031

[16] Sadeghi F Ardestani A Hadji M (2017). Travel Burden for Cancer Patients in Iran: Analysis of 1700 Patients from the Cancer Institute of Iran. Arch Iran Med, 20:147–152. 28287808

[17] Movahedi A Roosta R (2000). Food composition table. Shaheed Beheshti University of Medical Sciences Tehran, Iran

[18] Dorosti A Tabatabaei M (2007). Food composition table. The Iranian Nutrition World Journal, 16:15–20.

[19] Azar M Sarkisian E (1980). Food composition table of Iran. Tehran: National Nutrition and Food Research Institute, Shaheed Beheshti University, 65.

[20] Boushey CJ Coulston AM Rock CL Monsen E (2001). Nutrition in the Prevention and Treatment of Disease. ed. Elsevier.

[21] Rosner B Willett W (1988). Interval estimates for correlation coefficients corrected for within-person variation: implications for study design and hypothesis testing. Am J Epidemiol, 127:377–386. 3337089 10.1093/oxfordjournals.aje.a114811

[22] Block G Hartman AM (1989). Issues in reproducibility and validity of dietary studies. Am J Clin Nutr, 50:1133–1138. 2683721 10.1093/ajcn/50.5.1133

[23] Serra-Majem L Andersen LF Henríque-Sánchez P (2009). Evaluating the quality of dietary intake validation studies. Br J Nutr, 102:S3–9. 20100366 10.1017/S0007114509993114

[24] Block G Woods M Potosky A Clifford C (1990). Validation of a self-administered diet history questionnaire using multiple diet records. J Clin Epidemiol, 43:1327–1335. 2254769 10.1016/0895-4356(90)90099-b

[25] Johansson L Solvoll K Bjørneboe G-E Drevon CA (1998). Under-and overreporting of energy intake related to weight status and lifestyle in a nationwide sample. Am J Clin Nutr, 68:266–274. 9701182 10.1093/ajcn/68.2.266

[26] Marks GC Hughes MC van der Pols JC (2006). Relative validity of food intake estimates using a food frequency questionnaire is associated with sex, age, and other personal characteristics. J Nutr, 136:459–465. 16424128 10.1093/jn/136.2.459

[27] Ngo J Gurinovic M Frost-Andersen L Serra-Majem L (2009). How dietary intake methodology is adapted for use in European immigrant population groups–a review. Br J Nutr, 101:S86–94. 19594968 10.1017/S0007114509990614

[28] Black A Goldberg G Jebb S (1991). Critical evaluation of energy intake data using fundamental principles of energy physiology: 2. Evaluating the results of published surveys. Eur J Clin Nutr, 45:583–599. 1810720

